# Clinical, microbiological, and genomic characteristics of clade-III *Candida auris* colonization and infection in southern California, 2019–2022

**DOI:** 10.1017/ice.2022.204

**Published:** 2023-07

**Authors:** Annabelle de St. Maurice, Urvashi Parti, Victoria E. Anikst, Thomas Harper, Ruel Mirasol, Ayrton J. Dayo, Omai B. Garner, Kavitha K. Prabaker, Shangxin Yang

**Affiliations:** 1 Department of Clinical Epidemiology and Infection Prevention, Los Angeles, California; 2 Department of Pathology and Laboratory Medicine, UCLA David Geffen School of Medicine, Los Angeles, California

## Abstract

**Background:**

*Candida auris* is an emerging fungal pathogen causing outbreaks in healthcare facilities. Five distinctive genomic clades exhibit clade-unique characteristics, highlighting the importance of real-time genomic surveillance and incorporating genotypic information to inform infection prevention practices and treatment algorithms.

**Methods:**

Both active and passive surveillance were used to screen hospitalized patients. *C. auris* polymerase chain reaction (PCR) assay on inguinal-axillary swabs was performed on high-risk patients upon admission. All clinical yeast isolates were identified to the species level. *C. auris* isolates were characterized by both phenotypic antifungal susceptibility tests and whole-genome sequencing.

**Results:**

From late 2019 to early 2022, we identified 45 patients with *C. auris*. Most had a tracheostomy or were from a facility with a known outbreak. Moreover, 7 patients (15%) were only identified through passive surveillance. Also, 8 (18%) of the patients had a history of severe COVID-19. The overall mortality was 18%. Invasive *C. auris* infections were identified in 13 patients (29%), 9 (69%) of whom had bloodstream infections. Patients with invasive infection were more likely to have a central line. All *C. auris* isolates were resistant to fluconazole but susceptible to echinocandins. Genomic analysis showed that 1 dominant clade-III lineage is circulating in Los Angeles, with very limited intrahost and interhost genetic diversity.

**Conclusions:**

We have demonstrated that a robust *C. auris* surveillance program can be established using both active and passive surveillance, with multidisciplinary efforts involving the microbiology laboratory and the hospital epidemiology team. In Los Angeles County, *C. auris* strains are highly related and echinocandins should be used for empiric therapy.

*Candida auris* is a multidrug-resistant emerging fungal pathogen.^1^ It may be difficult to identify with standard laboratory methods, which may lead to misdiagnosis and increased transmission.^2^ Multiple healthcare-associated outbreaks of *C. auris* have occurred across the United States.^2,3^ From July 2021 to December 2021, the CDC identified 3,772 cases of colonized *C. auris* in the United States.^4^ Genomic epidemiology has characterized the global emergence of 5 major clades of *C. auris.* ^5^ Each clade is differentiated by their geographic origination. Clade I is from South Asia, clade II is from East Asia, clade III is from Africa, clade IV is from South America, and clade V is from Iran.^6,7^ These clades also exhibit unique clinical and microbiological characteristics. For instance, clade I has the highest frequency of antifungal resistance; clades I, III, and IV are frequently associated with outbreaks. Clade II, which has not been associated with outbreaks, is also less pathogenic and less drug resistant.^8,9^ Clade-unique characteristics highlight the importance of real-time genomic surveillance and incorporating *C. auris* genotypic information to inform infection prevention practices and treatment algorithms.

Antiseptic practice in the healthcare setting, agricultural antifungal usage, and high frequency of global travel have all been speculated to contribute to the emergence of these multidrug-resistant yeasts.^10,11^ In the United States, exposure to long-term acute-care hospital (LTACH) and skilled nursing facility (SNF) are risk factors for *C. auris* colonization and infection.^3,12^ We previously reported a community outbreak of genetically related clade-III *C. auris*, which started in late 2019 in the Los Angeles area. All isolates were resistant to fluconazole but susceptible to echinocandins, and all cases had exposure history to an LTACH or SNF.^13^ Based on our findings, we continued to perform active surveillance for high-risk patients for *C. auris* and perform real-time genomic surveillance to monitor the evolution of the outbreak. Here, we summarize the clinical, microbiological, and genomic characteristics of our *C. auris* cases from 2019 to 2022.

## Methods

### Candida auris *screening and isolation precautions*

**Fig. 1.**
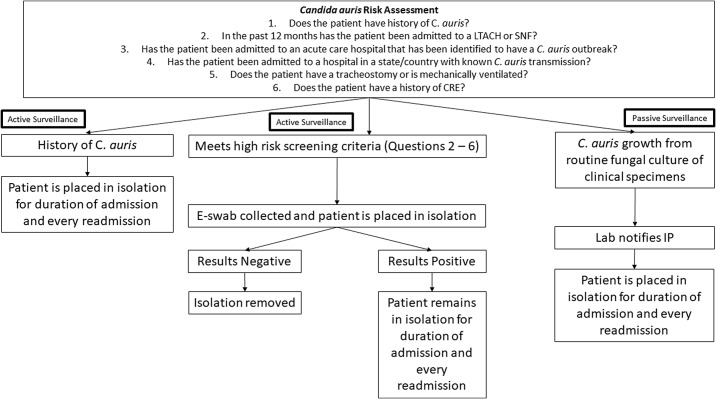
Workflow of the UCLA hospital *C. auris* screening program.

*Candida auris* screening at our institution includes both active and passive surveillance (Fig. 1). Beginning October 2019, all high-risk patients were screened for *C. auris* colonization using an in-house PCR test on composite swabs of bilateral axillae and groin collected using an ESwab (Becton Dickinson, Franklin Lakes, NJ).^13^ The DNA was extracted using the EZ1 Tissue Extraction kit on the EZ1 Advanced XL (Qiagen, Germantown, MD) and the polymerase chain reaction (PCR) was performed using the *C. auris* Primer Pair and Universal Master Mix on the LIAISON MDX (DiaSorin Molecular, Cypress, CA). The limit of detection of the PCR test was determined to be 100 colony-forming units per milliliter. In accordance with the Centers fo Disease Control and Prevention (CDC) and the Los Angeles County Department of Public Health (LACDPH) guidelines,^12,14^ high-risk patients included those admitted to a facility with a known *C. auris* outbreak, those who had an overnight hospitalization in a state or country with known *C. auris* transmission, those with a history of carbapenem-resistant Enterobacterales (CRE), those with a history of tracheostomy or mechanical ventilation, or whose who had been in contact with someone with *C. auris* colonization or infection. On September 1, 2021, the LACDPH recommended that acute-care hospitals screen all patients admitted from LTACHs and ventilator-capable SNFs (vSNFs). At our facility, we began screening patients who met criteria in the emergency department on July 14, 2021, and the program expanded to screen patients from all SNFs (including those without ventilator units) on September 2, 2021. Patients with a known history of *C. auris* were often screened upon admission; however, this was not mandatory because patients may be intermittently colonized.

**Fig. 2.**
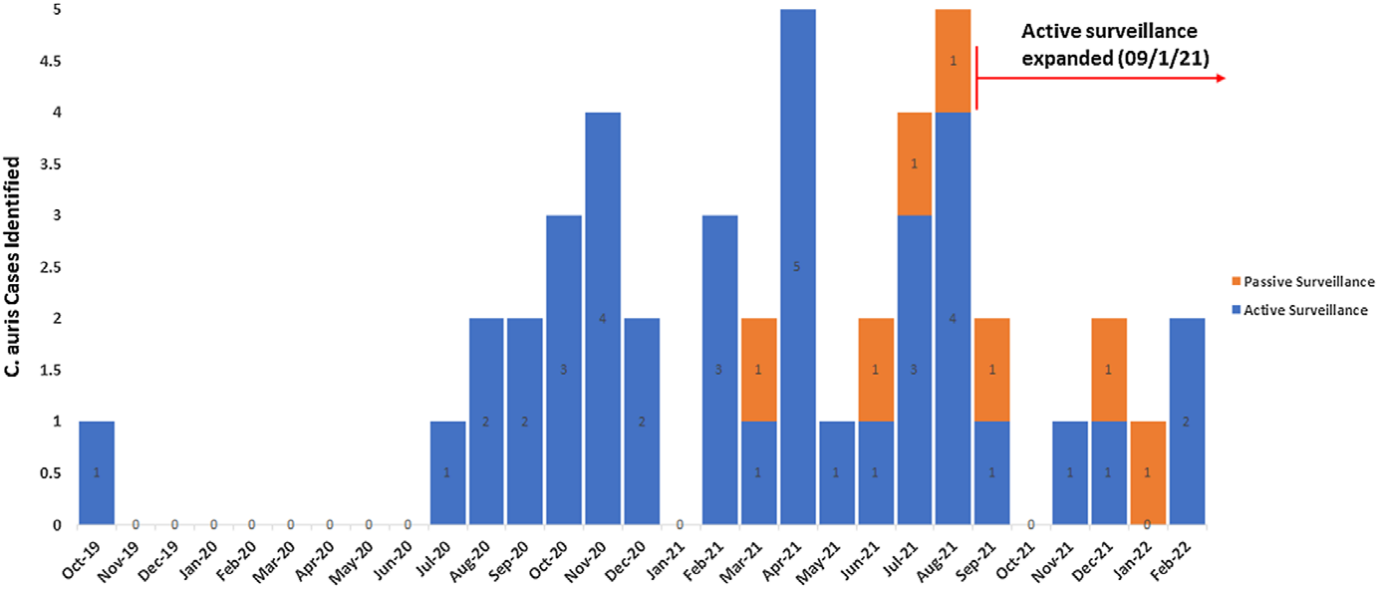
Timeline and positive *C. auris* cases identified by either active or passive surveillance.

Patients with suspected or known *C. auris* (eg, those awaiting *C. auris* test results) were immediately placed in contact/spore isolation. The environmental services staff was notified if a *C. auris* confirmed or suspect patient changed rooms or was discharged, and the room was then cleaned using a sporicidal disinfectant. Ultraviolet disinfection was performed, followed by cleaning validation with adenosine triphosphate (ATP).^15^

### Microbiological workup and passive surveillance

PCR-positive inguinal-axillary swabs obtained through active surveillance were inoculated onto the CHROMagar (Hardy Diagnostics, Santa Maria, CA) and were incubated at 35°C, ambient air, for up to 3 weeks. Passive surveillance was also implemented to identify to the species level all yeasts grown from clinical samples using a VITEK matrix-assisted laser desorption/ionization time-of-flight mass spectroscopy (MALDI-TOF MS) system (bioMérieux, Hazelwood, MO). Broth microdilution–based antifungal susceptibility testing was performed using panels prepared in house as previously described.^13^

### Genomic surveillance

Whole-genome sequencing and data analysis were performed as previously described.^13^ Genes implicated in antifungal resistance (ie, *erg11* for azole and *fks1* for echinocandin) were analyzed for mutations. The copy number of *erg11* gene was estimated using the ratio of the average mapping coverage of the single gene over the whole genome.

### Chart review and research ethics

We included all hospitalized *C. auris* cases at our healthcare system from October 1, 2019, to February 28, 2022. The electronic medical record was utilized to abstract clinical and laboratory information from the patient’s first hospitalization when *C. auris* was detected. To evaluate the efficacy of our screening, we excluded *C. auris* cases who did not have a prior history of *C. auris* and whose cases were not identified by PCR within 48 hours of admission. Immunosuppression included patients with primary or secondary immunodeficiencies. Cases with positive cultures were reviewed by 2 experienced infectious disease physicians (A.d.S.M. and K.P.) to determine whether they had a clinical infection.

This study has been reviewed by the UCLA Human Research Protection Program and was approved with an exemption from the institutional review board.

### Statistical analysis

Data were analyzed using STATA software (StataCorp, College Station, TX). The χ^2^ test, Fisher exact test, and Wilcoxon rank-sum test were used as appropriate.

## Results

### *Results of* C. auris *screening*

In 2.5 years (October 2019–February 2022), we identified 65 positive results among 1,380 *C. auris* screening PCR tests performed (4.7% positivity), which led to 45 *C. auris* cases of 1,129 unique patients screened (4.0% prevalence) (Fig. 2). Moreover, 32 patients were detected by screening PCR within 48 hours of admission. Of these 32 patients, 11 (34%) had a known history of *C. auris* and the remaining 21 were newly identified as being colonized with *C. auris.* Also, 9 cases were excluded because they did not have a known history of *C. auris* and were not detected by PCR within 48 hours of admission. Of these 9 cases, 4 did not meet criteria for screening on admission, 3 had indeterminate PCR results on admission, and 2 met the criteria for screening but screening was missed or delayed on admission.

Our passive surveillance system identified 7 cases through fungal culture that would not have been identified otherwise. Of these, 3 cases were identified by respiratory culture, 2 by blood culture, 1 by permacather tip culture, and 1 by urine culture.

### Demographics and clinical characteristics

Most patients with *C. auris* colonization or infection (60%) were male, and the median age of all patients was 66 years (Table 1). All 45 patients had at least 1 comorbidity; however, only 16% were immunosuppressed. Immunocompromising conditions identified included active malignancy or cirrhosis. Most patients had a history of tracheostomy (82%) or were from a facility with a known *C. auris* outbreak (89%). Facility A accounted for 32 (71%) of *C. auris* patients identified. Moreover, 8 (18%) of the patients had a history of severe COVID-19 within the past year, and 6 had respiratory failure due to COVID-19 at the time of *C. auris* colonization or infection. Mortality during the index *C. auris* hospitalization was 18%. Other demographic characteristics are listed in Table 1.

**Table 1.** Demographic Characteristics of *C. auris* Colonized Versus Infected Patients

Characteristic	Total (N=45)	*C. auris* Colonized (N=32, 71%)	*C. auris* Infected (N=13, 29%)	*P* Value
Sex, male, no. (%)	27 (60)	22 (69)	5 (38)	.094
Age, median y (IQR)	67 (57–75)	65 (57–77)	69 (59–73)	.86
Comorbidities, no. (%)	45 (100)	32 (100)	13 (100)	…
Immunosuppressed, no. (%)	7 (16)	5 (16)	2 (15)	1.0
Current or prior history of CRE, no. (%)	2 (4)	2 (6)	0	1.0
Current or prior history of MDRO, no. (%)	18 (40)	13 (41)	5 (39)	1.0
Current or prior history of invasive fungal infections, no. (%)	3 (7)	1 (3)	2 (15)	.19
**Diabetes mellitus, no. (%)^a^**	28 (62)	19 (59)	9 (69)	.54
Type I diabetes	0	0	0	
Type II diabetes	26 (58)	17 (53)	9 (69)	
Presence of any invasive device, no. (%)	43 (96)	30 (94)	13 (100)	1.0
Dialysis, no. (%)	7 (16)	3 (9.4)	4 (31)	.17
**Type of invasive device, no. (%)**				
Tracheostomy	37 (82)	27 (84)	10 (77)	.67
Gastrointestinal tube	36 (80)	27 (84)	9 (69)	.41
Central line	15 (33)	7 (29)	8 (62)	**.02**
Foley catheter	22 (49)	17 (53)	5 (39)	.51
Chest tube	4 (9)	1 (3)	3 (23)	.07
LTACH/SNF with known outbreak, no. (%)	40 (89)	30 (94)	10 (77)	.14
**LTACH/SNF/hospital, no. (%)**				
Facility A	32 (71)	26 (81)	6 (46)	
Facility B	5 (11)	3 (9.4)	2 (15)	
Facility C	1 (2)	0	1 (7.7)	
Facility D	2 (4)	1 (3.1)	1 (7.7)	
Recent COVID-19 history	8 (18)	5 (15)	3 (23)	.67
Hospital length of stay, median d (IQR)	13 (6–24)	10 (6–28)	16 (7–24)	.29
Mortality during hospitalization, no. (%)	8 (18)	5 (16)	3 (23)	.67
**Discharge location, no. (%)**				
Home	2 (5.7)	0	3 (23)	
SNF	27 (77)	23 (72)	5 (38)	
LTACH	2 (5.7)	1 (3.1)	1 (7.7)	
Acute-care hospital	3 (8.6)	2 (6.2)	1 (7.7)	
Hospice	1 (2.9)	1 (3.1)	0	

Note. IQR, interquartile range; CRE, carbapenem-resistant Enterobacterales; MDRO, multidrug-resistant organism; LTACH, long-term acute-care hospital; SNF, skilled nursing facility.

a In some instances type of diabetes was unspecified in the chart.

Of the 45 patients identified with *C. auris*, 18 (40%) had positive cultures for *C. auris*. Among the 18 patients with clinical cultures growing *C. auris*, 13 patients (72%) were categorized as having a clinical infection (Table 2). Of 13 patients with clinical infections, 11 were treated with an echinocandin alone and 1 patient was treated with a combination of echinocandin and liposomal amphotericin. Also, 1 patient died prior to their blood culture growing *C. auris* and was therefore untreated.

**Table 2.** Characteristics of Patients (n=13) with Invasive *C. auris*

Characteristic	No. (%)
Comorbidities	13 (100)
Chronic respiratory failure	8 (61)
End-stage renal disease	4 (31)
Diabetes	9 (69)
Cirrhosis	1 (8)
Malignancy	1 (8)
Known history of *C. auris* prior to infection	8 (62)
Received treatment for *C. auris* infection	12 (92)^a^
***C. auris*** **antifungal treatment**	
Caspofungin	11 (85)
Anidulafungin	1 (8)
Liposomal amphotericin^b^	1 (8)
**Site of infection**	
Blood	9 (69)
Urine	3 (23)
Pleural fluid	3 (23)
Tracheal aspirate	2 (15)
Wound	2 (15)

a For 1 patient, the blood culture grew *C. auris* after the patient died; therefore, the patient did not receive treatment.

b One patient received combination therapy with caspofungin and liposomal amphotericin.

When comparing the patients who had clinical infection to those who were colonized with *C. auris*, patients who had a clinical infection were significantly more likely to have a central line (Table 1). The mortality rates during the index hospitalization between the colonized and infected patients were not significantly different.

### Microbiological characteristics and antifungal susceptibility patterns

**Fig. 3.**
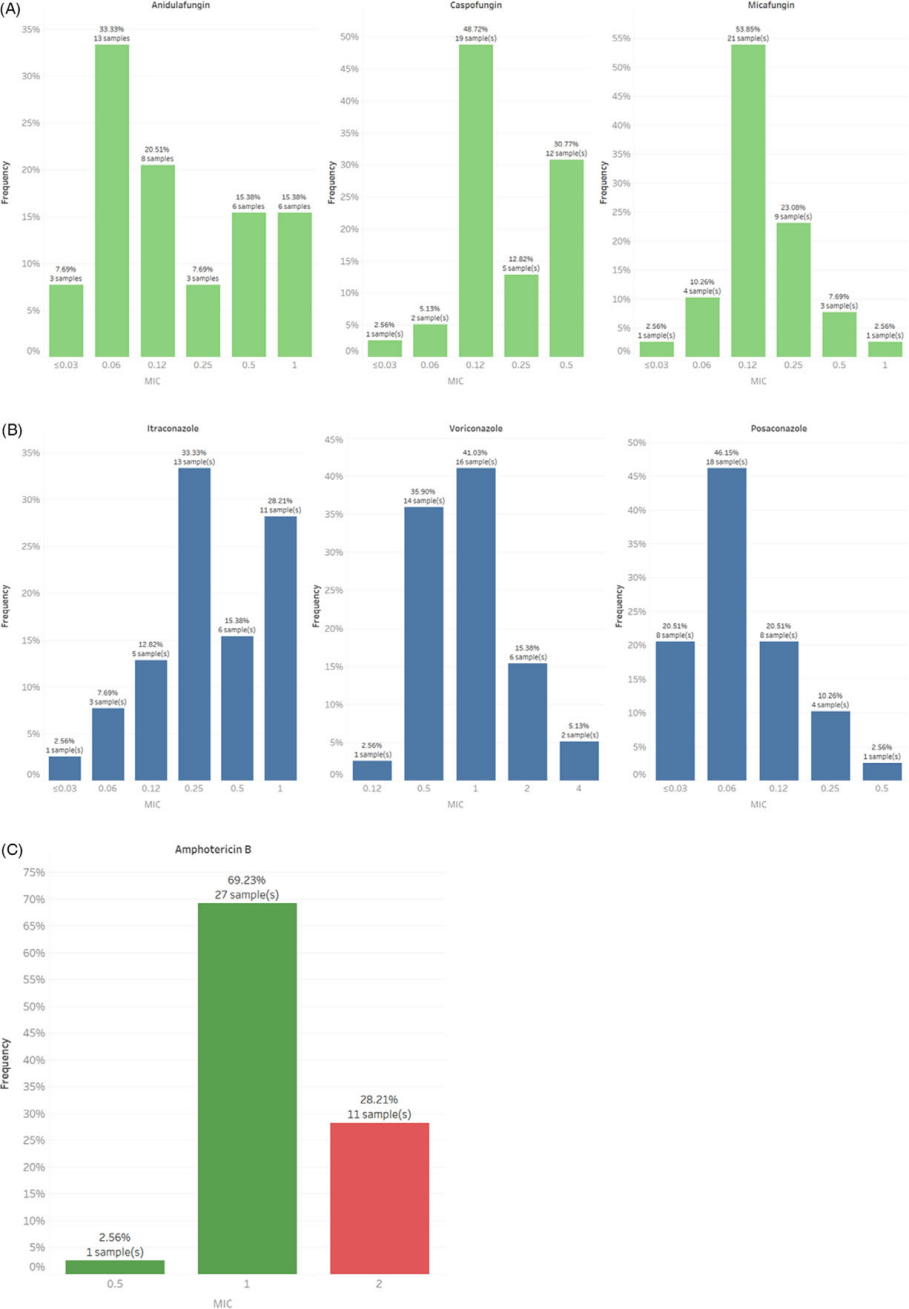
Summary of antifungal susceptibility results and MIC distribution for echinocandins (3A), azoles (3B) and amphotericin B (3C). Note: We adopted the CDC tentative MIC breakpoints for *C. auris*: amphotericin B (≥2 µg/mL), fluconazole (≥32 µg/mL), anidulafungin (≥4 µg/mL), caspofungin (≥ 2µg/mL), micafungin (≥4 µg/mL). Voriconazole, itraconazole, and posaconazole were also tested but without interpretative criteria.

*C. auris* was isolated from only 19 (45%) of 42 PCR-positive inguinal or axillary swabs, indicating a low sensitivity of regular fungal culture for *C. auris* screening without enrichment medium such as dulcitol broth.^16^ Antifungal susceptibility testing was performed on 39 isolates from 28 patients, including 18 patients with infections and 10 patients with colonization alone. All isolates were resistant to fluconazole with MIC ≥ 64 mg/mL but were susceptible to echinocandins. The minimum inhibitory concentrations (MICs) ranged from ≤0.03 to 1 for anidulafungin, from ≤0.03 to 0.5 for caspofungin, and from ≤0.03 to 1 for micafungin (Fig. 3A). For other azoles without Clinical and Laboratory Standards Institute (CLSI) break points, the MICs ranged from 0.12 to 4 for voriconazole, from ≤0.03 to 1 for itraconazole, and from ≤0.03 to 0.5 for posaconazole (Fig. 3B). For amphotericin B, 11 (28%) of 39 isolates were considered resistant (Fig. 3C).

### Genomic characteristics

**Fig. 4.**
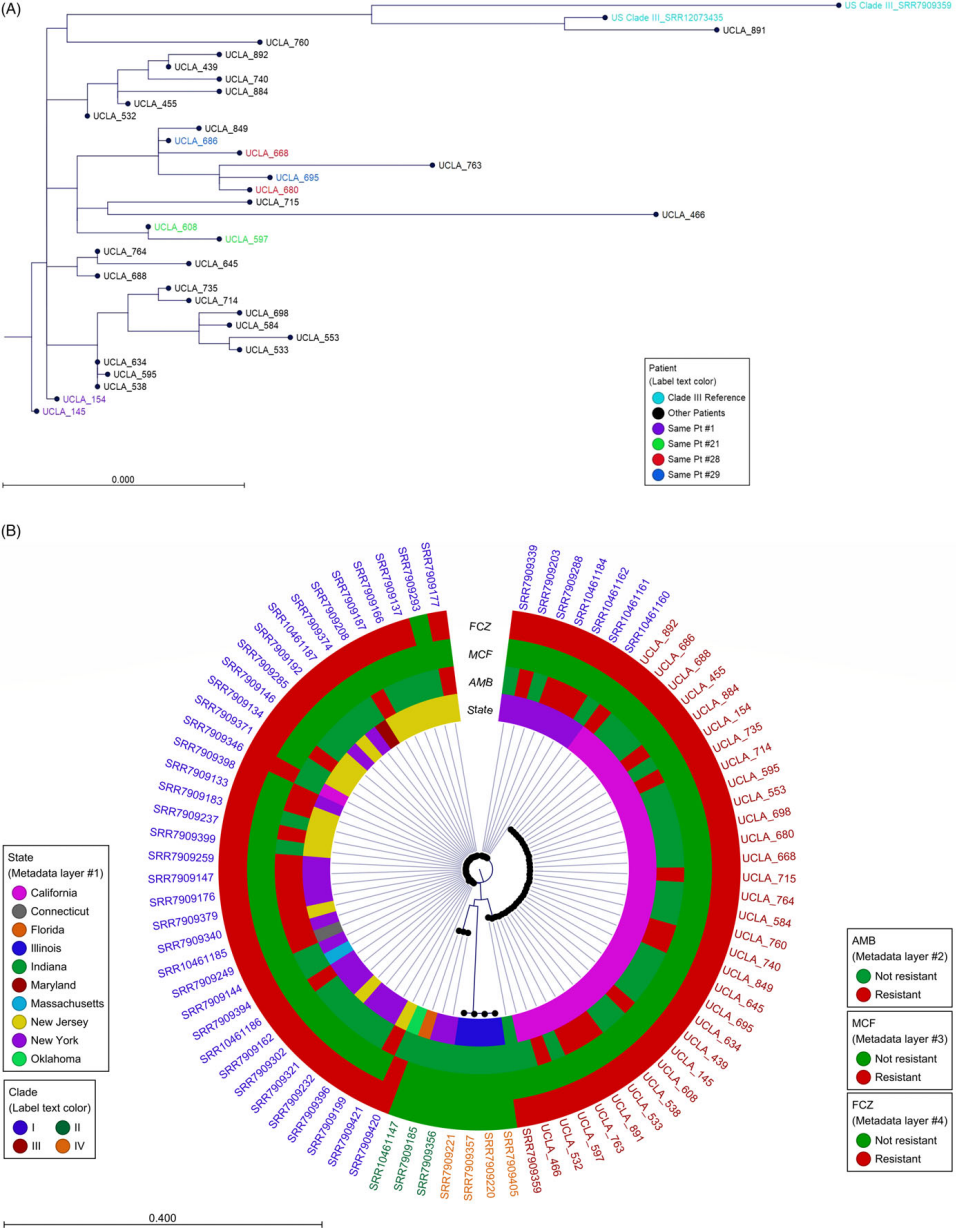
(A) SNP phylogenetic tree of the UCLA *C. auris* isolates. (B) *K-mer* phylogenetic tree of *C. auris* in the United States by states and clades.

Overall, 32 isolates from 28 patients (18 infected and 10 colonized) were sequenced, with at least 1 isolate per patient and 2 isolates in 4 patients. Sufficient sequence reads (1,392,156–6,212,112; median 2,588,576) were acquired for each isolate, resulting in >90% whole-genome coverage (91.15%–97.75%; median, 97.67%) with at least 10× depth in all isolates. All of the UCLA *C. auris* isolates were in the clade III. Except for UCLA-466 and UCLA-891, all other isolates clustered closely together, with only 2–50 single-nucleotide polymorphisms (SNPs) (Fig. 4A and Supplementary Fig. S1 online), which indicated a single origin.^13^ They are loosely related to other domestic clade-III *C. auris* stains, including SRR7909359 isolated in a patient in Indiana in 2017 and SRR12073435 found in Florida in 2019 (Fig. 4B), with 69–99 SNP differences and 47–77 SNP differences, respectively (Supplementary Fig. S1 online). UCLA-466 is genetically distinctive, with 73–97 SNPs compared to all other UCLA isolates, and it branched separately in the SNP tree (Fig. 4A). It is also less related to the Indiana and the Florida *C. auris* strains, with 135 and 122 SNPs, respectively, suggesting a different entry without a known origin. UCLA-891 (isolated in February 2022) clustered closely with the Florida strain with only 21 SNPs, which is consistent with the history that this patient was transferred from a healthcare facility in Florida in late 2021. Notably, in 4 patients, we sequenced 2 isolates per patient from samples collected at different sources within 10 days, and the results showed very limited intrahost genetic diversity, with only 2–11 SNP difference.

In the *erg11* gene, all isolates possess the 2 mutations, p.V125A and p.F126L, which are well known for causing fluconazole resistance in clade-III *C. auris* (Supplementary Table S1 online).^5,13^ Except for UCLA-891, which is related to the Florida strain, all other isolates did not have increased gene copy number, which had been reported in a small percentage of the global clade-III *C. auris*.^5^ UCLA-891 possessed 2 copies of the *erg11* gene, indicating an additional azole resistance mechanism. However, its phenotypic azole resistance profile is not different from other isolates, with an MIC of >64 for fluconazole, an MIC of 1 for voriconazole, an MIC of 0.5 for itraconazole, and an MIC of 0.06 for posaconazole. In the *fks1* gene, all isolates have a p.I1572L polymorphism that is not associated with antifungal resistance. One isolate (UCLA-466) carries an additional polymorphism p.I1095L, consistent with its unique phylogeny indicating a separate entry. No mutations were identified at S639, which had been linked to echinocandin resistance.^5^

## Discussion

In this study, we have described a robust surveillance system established by a multidisciplinary team consisting of clinical microbiologists, infectious diseases specialists, environmental services, informational technology specialists, and clinical epidemiologists. In this system, *C. auris* cases of colonization and infection were effectively identified using a combination of active screening by a highly sensitive PCR test and passive surveillance relying on accurate species-level identification for all yeasts grown from clinical samples. We identified our first *C. auris* case as early as October 2019. From November 2019 to June 2020, no cases were identified by either active or passive surveillance. However, since July 2020, more cases have been identified, with a peak during the second and third quarters of 2021, with as many as 5 cases identified monthly.

In this study, 29% of patients identified through surveillance had *C. auris* infection. Comorbidities were present in all patients with *C. auris,* including patients who were colonized; however, infected patients were more likely to have a central line. Most of the clinical infections identified were bloodstream infections, which is similar to the findings of other studies.^1^ Interestingly, although half of infected *C. auris* patients developed fungemia in our study, mortality did not differ significantly between *C. auris* infected versus colonized patients, likely due to the small number of cases. The overall mortality rate in our patient population was 18%, which is lower than the 30%–60% mortality rates reported in other studies^17^; this rate is likely low because we were conducting active surveillance. As our screening criteria expands to include patients admitted from all SNFs and LTACHs, we will likely find more patients colonized with *C. auris* outside known outbreak facilities, and potentially we will identify new facilities with *C. auris* outbreaks.

We identified most patients during the COVID-19 pandemic. The interaction between severe acute respiratory coronavirus virus 2 (SARS-CoV-2) and *Candida* infections has been documented. In one study, patients with *Candida* and SARS-CoV-2 coinfection often did not have typical candidemia risk factors, but high healthcare utilization during the pandemic likely contributed to their infections; unfortunately, this study did not include patients with *C. auris.* ^18^ A different study of patients infected with *C. auris* during the pandemic in India reported that the use of tocilizimab, duration of ICU stay, and high ferritin level were predictors of candidemia.^19^ Among our patients, 8 (18%) had histories of severe COVID-19 with prolonged hospitalization. Reuse of PPE due to supply shortages, increased antimicrobial use, and prolonged hospital stay may have contributed to the spread of *C. auris* within healthcare facilities, precipitating outbreaks during the pandemic.^20,21^ Additionally, some healthcare facilities placed patients in cohorts based on COVID-19 status alone before patients were identified as being colonized with multidrug-resistant organisms.^21^ Timely screening and identification of patients colonized with *C. auris* can improve isolation measures and help prevent the spread of *C. auris* within an institution.

Despite following the CDC and LACDPH guidelines for active screening of the patients with high risk, 4 patients (9%) did not meet screening criteria on admission, suggesting increasing community spread which rendered the selected screening scheme less effective. Universal screening may become increasingly important as spread continues; however, universal screening may still miss cases. In our study, 3 patients had indeterminate PCR results and 1 patient had a negative PCR but later developed infection. We later modified our active surveillance process to repeat PCR screening with a new swab rather than repeat PCR on the original swab to increase sensitivity of our active surveillance. Further research should be conducted to determine the most sensitive methods to test for *C. auris* colonization. For instance, culture using dulcitol enrichment broth has shown high sensitivity for effectively isolating *C. auris* from patients and their environment.^16^ Among our patients, 7 (16%) were only identified through passive surveillance, which highlights the importance of continuing passive surveillance in parallel with active screening.

To our knowledge, this is the first study to characterize many clade-III *C. auris* cases in the United States. Our genomic analysis showed that only 1 dominant and unique lineage has been circulating in the LA area in the past 2.5 years. Very limited genetic diversity was observed in the general patient population and within hosts. We also showed a recent interstate transmission of *C. auris* that was introduced from Florida to southern California, which demonstrates the importance of screening patients recently admitted from facilities in high-prevalence states. All the isolates identified so far were resistant to fluconazole but susceptible to echinocandins. The interpretation of the amphotericin B MIC results in *C. auris* remains challenging due to a lack of method standardization. The consistent drug-susceptibility pattern for azoles and echinocandins, and the limited genetic diversity of the *C. auris* outbreak strain characterized in this study, can serve as reliable evidence to guide effective empiric treatment in Los Angeles, that is, using echinocandins as the first-line antifungal drug for treating *C. auris* infections.

This study had several limitations. Data were collected retrospectively; thus, some of the information about patient-level risk factors was incomplete, including travel. A standard definition for *C. auris* colonization versus infection is lacking. This study was performed at a single institution, and 71% of our patients with *C. auris* came from a single facility; therefore, our *C. auris* isolates may not cover all the community strains in Los Angeles. Very limited genes known to be associated with antifungal resistance were analyzed, which highlights an urgent need for more expansive understanding of molecular mechanisms for drug resistance in *C. auris*. Adherence to our screening guidelines was not consistent; 2 patients met screening criteria but did not have a screening PCR sent on admission. Missed screening opportunities could be partially due to staffing challenges during the COVID-19 pandemic. In addition, we missed 1 screening opportunity due to a patient being directly admitted from a low-risk facility; however, this patient had been admitted to a high-risk facility within the prior 12 months and should have been screened. Admission history is difficult to obtain from chronically ill patients, many of whom may be nonverbal and may not be accompanied by family members.

In summary, we have demonstrated that a robust *C. auris* surveillance program can be established with multidisciplinary efforts involving both the microbiology laboratory and the hospital epidemiology team. Our study has provided real-time and critical information about the characteristics of the outbreak *C. auris* strain that can be used to guide effective treatment policies. This type of screening does require significant institutional support because screening is not reimbursed by insurance and may be perceived as low yield given the low percentage of patients who test positive for *C. auris*. However, given our high-risk population, this type of active surveillance plays an instrumental role in controlling the spread of *C. auris* in the hospital setting.

## Supplementary material

For supplementary material accompanying this paper visit https://doi.org/10.1017/ice.2022.204. click here to view supplementary material
